# Characteristics of inpatient and outpatient respiratory syncytial virus mortality in Gavi-eligible countries

**DOI:** 10.1016/j.jvacx.2024.100554

**Published:** 2024-09-13

**Authors:** Joukje E. Willemsen, Femke S. Vernooij, Farina L. Shaaban, Chilufya Chikoti, Louis J. Bont, Julia Drylewicz, the RSV GOLD study group

**Affiliations:** a Centre for Translational Immunology, University Medical Centre Utrecht, Utrecht, The Netherlands; b Division of Infectious Diseases, Department of Pediatrics, University Medical Centre Utrecht, Utrecht, The Netherlands; c Right to Care Zambia, Lusaka, Zambia

**Keywords:** RSV, Gavi-eligible countries, Age at RSV-related death, Mortality

## Abstract

The current understanding of the RSV-related mortality age distribution in low- and lower-middle-income countries (LMICs) relies on a limited number of disease incidence studies reporting wide age bands, and lacking specificity to Gavi-eligible countries. Understanding the age distribution of RSV-related deaths is crucial for the implementation of RSV interventions in LMICs that rely on support from Gavi. This study aims to provide the age profile of RSV mortality specifically in Gavi-eligible countries.

Utilizing data from the RSV GOLD project, an ongoing global online mortality registry focusing on children under the age of 5 with laboratory-confirmed RSV infection, we employed two models (Complete Data Model and Prospective Data Model) to estimate the age profiles. To mitigate biases related to age group representation, we applied post-stratification weighting in our analysis.

We included 423 pediatric deaths, including 145 from the community, under 2 years of age from 15 Gavi-eligible countries. Both models identified a peak age at 1 month and found that the majority of RSV-related mortality cases (59–77 %) from Gavi-eligible countries occur before 6 months of life. However, the models exhibited disparities in other age-related metrics. We present fitted age-at-time-of-death probability distributions to aid impact and cost-effectiveness studies.

We expect that implementing infant RSV immunization strategies, such as maternal vaccination or infant immunoprophylaxis, will have high impact on RSV-related mortality in Gavi-eligible countries. The divergent results from the two models underscore the importance of carefully considering potential biases in retrospective and surveillance data when interpreting the age profile of RSV mortality cases in future research.

## Highlights

• Median age at RSV-related death in Gavi-eligible countries is lower than prior estimates.
• The majority of RSV-related deaths from Gavi-eligible countries occurs before 6 months of life.
• Presence of biases in retrospective data should be considered for cost-effectiveness analyses.

## 1 Introduction

Respiratory syncytial virus (RSV) infection is a leading cause of hospitalization and mortality worldwide in children under 5 years of age due to lower respiratory tract infection (LRTI) [1]. The majority of deaths (97 %) occur in low- and lower-middle-income countries (LMICs) due to the poor accessibility and affordability of healthcare and poor quality of care in health facilities. Gaining insight into the age distribution of children who experience fatal outcomes due to RSV has become more pressing with ongoing research into prevention methods, such as vaccines and monoclonal antibodies, and policy debates on prophylaxis in resource-limited settings [2].

Several RSV intervention products are currently in clinical development, and in 2023, both an RSV maternal vaccine and a long-acting single-dose monoclonal antibody (mAb) have received market approval. However, due to limited resources, maternal vaccination and mAb access in LMICs will require support from international partners [3]. Gavi, the Vaccine Alliance, is an international organization established with the goal of creating equal access to new and underused vaccines for those living in LMICs. To increase the accessibility of RSV prevention methods worldwide, Gavi has included both maternal vaccination and monoclonal antibodies for the prevention of RSV as one of the six priorities in their Vaccine Investment Strategy (VIS) for the 2021–2025 period [4], [5]. Every 5 years, the VIS sets new priorities for Gavi’s vaccine support programmes based on impact, cost, value and programmatic feasibility of underused or new vaccines of highest relevance to Gavi-eligible countries [6].

As both maternal vaccination and RSV monoclonal antibodies are characterized by a temporary protection profile, a good understanding of the age distribution of RSV-related deaths in Gavi-eligible countries will contribute to inform on impact and cost-effectiveness of RSV prophylaxis, thereby aiding Gavi in their decision-making process. The most commonly used estimates for the RSV-related mortality age-distribution in LMICs originate from a systematic review [1], [7]. This study relied on a limited number of studies centered on disease incidence and mortality rates, which were often presented in broad age categories, producing indirect estimates. Furthermore, these estimates were not specific to Gavi-eligible countries. The WHO Strategic Advisory Group of Experts on Immunization (SAGE) underlined the key epidemiological gap of age-stratified data on RSV acute lower respiratory infection (ALRI) during the first months of life to inform the use and anticipated impact of prevention products [5], [4]. Utilizing more detailed age-distributions enables a more precise estimation of the potential impact of RSV prevention strategies [8].

To fill the epidemiological gap of age-stratified disease burden data, the RSV Global Online Mortality Database (RSV GOLD) has been initiated in 2017, to elaborate on the clinical and sociodemographic profile of global RSV-related pediatric mortality. The first RSV GOLD retrospective case study was published in 2017 and included 117 cases from LMICs [9]. In 2021, a new retrospective case study was published comparing RSV-related infant community deaths with in-hospital deaths, including 829 deaths from LMICs [10]. However in that study, the majority of cases were from studies not eligible for Gavi, and the analysis on children below 2 years of age excluded 168 cases from community studies that only enrolled children up to 6 months of age.

In this study, we aim to investigate the characteristics of pediatric RSV mortality specifically in Gavi-eligible countries using all the data available from the RSV GOLD database. The detailed information on the age profile of RSV mortality will improve vaccine impact models and cost-effectiveness analysis, which are important for Gavi decision making.

## 2 Methods

### 2.1 Study design and patients

The RSV GOLD project is an ongoing global online mortality registry that collects individual patient data of children below 5 years of age who died with a laboratory-confirmed RSV infection after January 1st, 1995. Individual patient-level data are collected using an online questionnaire. Variables collected in the RSV GOLD database have been published previously [9], [10]. In this article we categorize the data in the RSV GOLD database into two groups based on how the information was collected. The characteristics (country, setting, inclusion criteria, etc.) of all the studies included in the RSV GOLD database are presented in STable 1. We refer to the first as Registry Mortality Data, which comprises data gathered through proactive outreach to researchers and physicians worldwide. These data were predominantly collected as part of *larger hospital-based surveillance studies*, mostly focused on respiratory infections in children. The sources are diverse, with variations in methodology and inclusion and exclusion criteria (see STable 1 for a detailed overview). The second group, referred to as Prospective Mortality Studies Data, consists of data from Bill & Melinda Gates Foundation (BMGF)-funded community mortality studies. These data were collected through *prospective hospital and community-based mortality surveillance*. Investigators of Bill & Melinda Gates Foundation (BMGF)-funded community mortality studies were specifically asked to share data collected up until March 2nd, 2021, as described previously in [10]. Two community studies (the Zambia Pertussis RSV Infant Mortality Estimation Study (Z-PRIME), and the Pakistan Community Mortality studies) included children younger than 6 months of age; other studies recruited children up until at least 12 months of age.

**Fig. 1:**
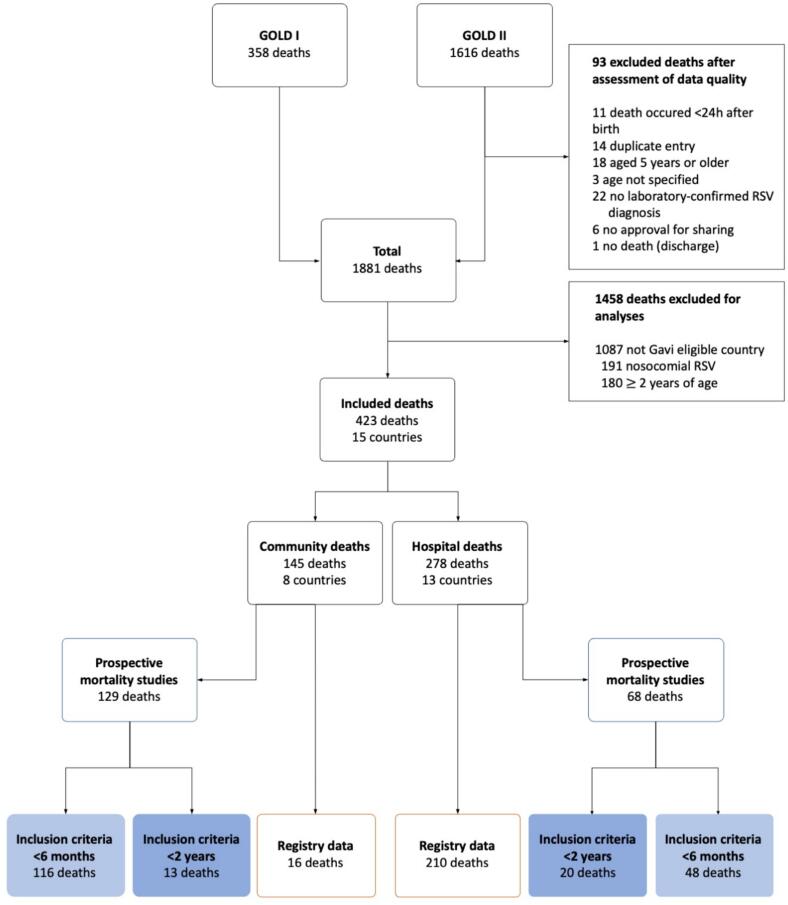
Flowchart of mortality cases included in this study. Flowchart shows children excluded via both data quality and per definition of study population. We incorporated the data from the <6 month and <24 months studies in our analysis using post-stratification weighting to adjust for overrepresentation. GOLD I: Pediatric deaths published as a retrospective case series from 1 November 2014 to 31 October 2015 [9]. GOLD II includes pediatric deaths collected after this publication. Abbreviations: m, months; BMGF, Bill & Melinda Gates Foundation; GOLD, Global Online Mortality Database; RSV, respiratory syncytial virus; ZPRIME, Zambia Pertussis RSV Infant Mortality Estimation Study.

All data were thoroughly reviewed by the RSV GOLD research team. Additional queries concerning inconsistencies or missing information have been verified through direct contact with the respective collaborator. In this analysis, RSV-related deaths above 2 years of age, nosocomial deaths, and deaths in high-income countries were excluded (Fig. 1).

### 2.2 Case definition

For this analysis, we only included mortality cases from Gavi-eligible countries according to the classification from 2023 [11]. As in our previous publications, we included any death with laboratory-confirmed RSV infection and did not require RSV to be the primary cause of death. If there was no information on where the RSV infection had been acquired and in the absence of nosocomial indications, we deemed the case community acquired.

The definition for a community death has been described previously [4], [8], [10]. If a child had not been admitted to hospital, we considered it as a community death. In case of missing data on the place of death, cases were classified as in-hospital deaths if the child had been admitted to hospital or if hospitalization status was not available. In case of missing data on hospitalization, we assumed the child had been hospitalized.

Children with comorbidities had at least one underlying disease, such as congenital heart disease, a genetic or chromosomal disorder, HIV infection, or active tuberculosis. Healthy term children were born without comorbidities at 37 weeks' gestational age or later, and healthy preterm children were born without comorbidities before 37 weeks' gestational age. If data for comorbidities and prematurity were not recorded, we assumed that the child was born healthy term.

### 2.3 Post-stratification weighting

In the RSV GOLD database, two prospective community mortality studies (Z-PRIME and the Pakistan Community Mortality studies) included only children younger than 6 months of age, while the vast majority of the registry mortality data included data until at least 2 years of age (STable 1). To address a potential issue of over- or under-representation of particular age groups in our analysis, we employed post-stratification weighting. We adjusted the weights of undersampled and oversampled subpopulations with the goal of making the overall sample more representative of the true underlying population. The weights were calculated based on the demographic characteristics of the known population, to which we refer as the population weights. In our case, the oversampled subpopulation was the group of children in Gavi-eligible countries who died with RSV before 6 months of age. We therefore needed to adjust the proportion of children in Gavi-eligible countries dying with RSV before 6 months of age and the proportion of children dying with RSV at 6 months or older. In order to estimate population weights based on our available data, we utilized two distinct approaches referred to as the Complete Data Model and the Prospective Data Model. Without a compelling reason to favor one model over the other, we examined the results of both models side by side.

**Fig. 2:**
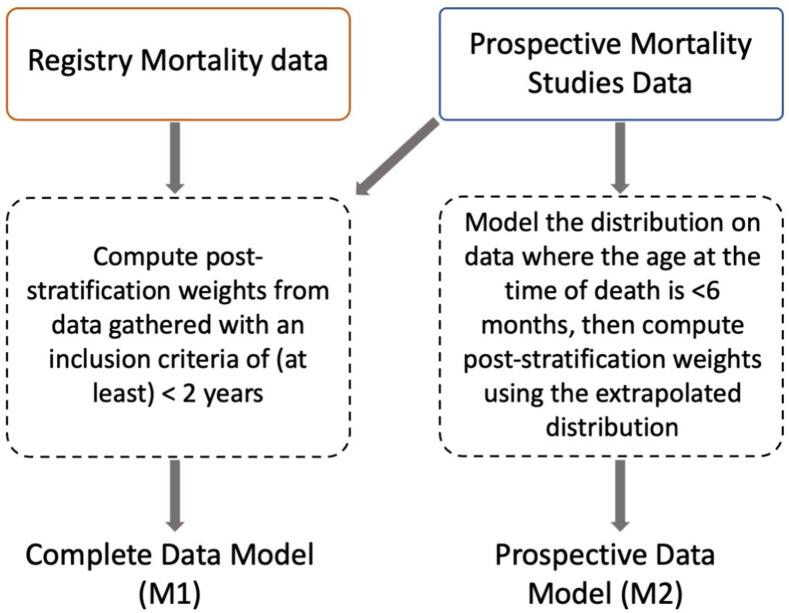
Visual summary of the difference between the Complete Data Model (M1) and the Prospective Data Model (M2). See SFig. 1 for a more detailed version.

In short, the Complete Data Model (M1) includes both the Registry Mortality Data and the Prospective Mortality Studies Data, whereas the Prospective Data Model (M2) includes only the Prospective Mortality Studies Data (Fig. 2). A complete illustrative comparison depicting the disparities between the Prospective Data Model and the Complete Data Model is presented in SFig. 1. More details about the models are described in Supplemental Methods.

### 2.4 Statistical analysis

We report the mean age at time of death, the median age at time of death (i.e. the age at which half of the deaths among those under 2 years old had already taken place), and the peak age at time of death (i.e. the age at which RSV-related mortality reaches its peak). To quantify the proportion of cases in age groups, which is relevant for developing prophylactics, we calculated the proportion of cases below 1, 3 and 6 months of age.

For the other clinical features of interest, we calculated descriptive statistics, including measures such as mean, standard deviation, median, mode and interquartile range. Categorical variables were presented as frequencies and percentages.

We used a general linear model with the “glm” function in R to investigate the relationship between the place of death (in-hospital or community) and the data collection methodology (registry or prospective) and the age at time of death.

### 2.5 Ethical approval

Since solely secondary anonymous data were used in this study, the Medical Ethics Review Committee at the University Medical Centre Utrecht waived parental informed consent. However, adherence to local guidelines was encouraged and collaborators obtained ethical approval whenever necessary.

### 2.6 Role of the funding source

The RSV GOLD study is funded by the BMGF, which had no role in the study design, data collection, analysis, and interpretation, or the writing of the article.

## 3 Results

### 3.1 Study population

**Fig. 3:**
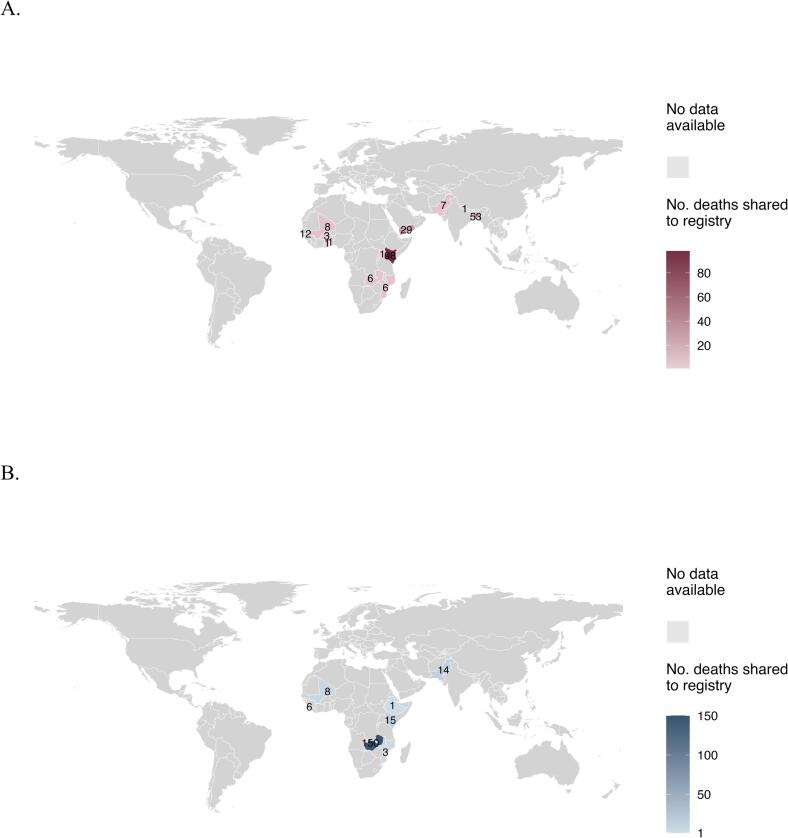
Countries of origin of included children with RSV-related mortality for the Registry Mortality Data (A) and the Prospective Mortality Studies Data (B), reference data for M1 and M2 respectively. The color gradient indicates the number of deaths shared, with a darker color representing a larger number of deaths shared.

We included 423 pediatric deaths under 2 years of age from 15 Gavi-eligible countries classified as LMIC according to the World Bank income group classification of 2023. Of these, 145 deaths occurred in the community (Fig. 1). The Registry Mortality Data consisted of 226 deaths (Fig. 1) from 13 different countries (Fig. 3A), of which 16 were community mortality cases and 210 were in-hospital mortality cases. Place of death was missing for 35 cases, hospitalization was missing for 2 cases.

**Fig. 4:**
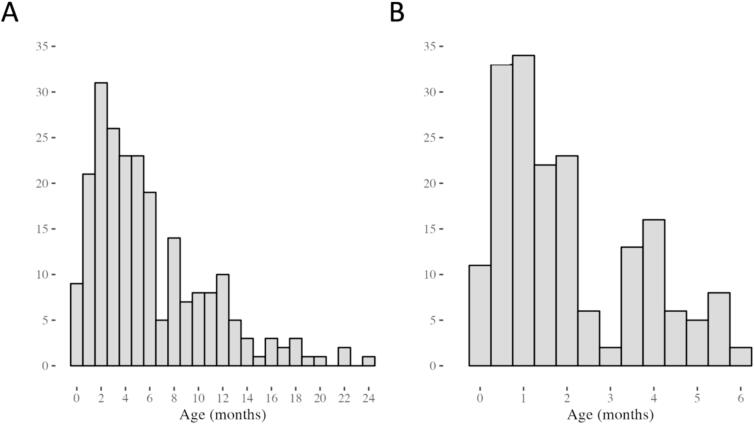
(A) Histogram of age at RSV-related death for children under 2 years in the Registry Mortality Data (reference data for M1). (B) Histogram of age at RSV-related death for children under 2 years in the Prospective Mortality Studies Data (reference data for M2). The histograms show the number of deaths (count) shared to the registry by age at death in months (rounded to the nearest integer).

RSV diagnosis was established most often by PCR (76 %). PCR was mostly used for children who died after 2005. The age at time of death distribution for the Registry Mortality Data is plotted in Fig. 4A. The Prospective Mortality Studies Data consisted of 197 deaths (Fig. 1) from 7 different countries (Fig. 3B), of which 129 were community mortality cases and 68 were in-hospital mortality cases. RSV diagnosis was established by PCR for all these cases. Most deaths were from Zambia (76 %, 150/197). The age at time of death distribution for the Prospective Mortality Studies Data is plotted in Fig. 4B.

### 3.2 Pooling hospital and community cases

Given the limited number of community mortality cases in the registry data, and the limited number of in-hospital mortality cases in the prospective data, we conducted a permutation test to determine whether it is appropriate to combine the hospital and community mortality cases for the analyses. We computed the difference in medians through 1000 permutations, comparing permuted differences to the observed one to calculate a p-value. The test showed no significant median age difference between community and in-hospital mortality cases (p = 0.69 for the registry data and p = 0.67 for the prospective data below 6 months). We therefore concluded that these cases could be pooled in the main analysis. Similarly, we tested whether the observations below 6 months of age differed significantly between the registry and prospective group. The permutation test did not yield a statistically significant result (p = 0.44), indicating that pooling of the data was acceptable.

### 3.3 Post-stratification weights

#### 3.3.1 Complete data model (M1)

In this model, we made the assumption that using all available data, except for the Z-PRIME and Pakistan data, which had divergent age inclusion criteria, would provide us with the most accurate representation of the true population. This data encompasses observations across the entire age spectrum up to 2 years. Based on this data, we estimated the true proportion of mortality cases below 6 months of age to be 0.59. This corresponds to a weight of 0.788 for cases before 6 months of age and a weight of 1.633 for 6 months and older.

#### 3.3.2 Prospective data model (M2)

**Fig. 5:**
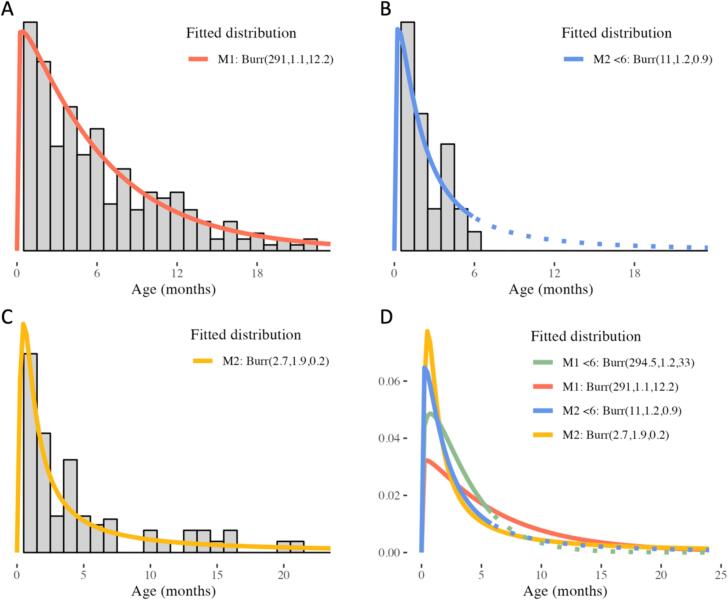
Fits of the Burr distribution to the age at RSV-related death data. The Burr distribution (Burr type XII) has three parameters: scale, shape 1 and shape 2. The fitted parameters are presented in the plot legend as Burr (scale, shape 1, shape 2). The cumulative distribution function of the Burr distribution (for *x* weeks of age) is $F\left(x\right)={1-[1+{\left(\frac{x}{scale}\right)}^{shape\;1}]}^{-shape\;2}$. (A) Weighted histogram (in grey) and fitted distribution (in red) for M1 under 2 years of age. (B) Data from the Prospective Community Mortality Studies below 6 months of age (in grey) and the corresponding fitted truncated distribution (in blue). (C) Weighted histogram (in grey) for M2 under 2 years of age and the fitted distribution (in yellow). (D) Comparison of the fitted distributions for M1 (in red the Burr-distribution fitted on the full dataset, in green the fitted Burr-distribution on data below 6 months of age only) and M2 (in yellow the Burr-distribution fitted on the weighted dataset, in blue the fitted Burr-distribution on data below 6 months of age only). (For interpretation of the references to color in this figure legend, the reader is referred to the web version of this article.)

In this model, we assumed that the subset of data obtained from the Prospective Community Mortality Studies is the most informative as this data was collected through active surveillance. We fitted a truncated Burr distribution (type XII) to the data below 6 months of age. The Burr distribution (Burr type XII) has three parameters [12]; scale, shape 1 and shape 2. The cumulative distribution function of the Burr distribution (for *x* weeks of age) is $F\left(x\right)={1-[1+{\left(\frac{x}{scale}\right)}^{shape\;1}]}^{-shape\;2}$. Our analysis identified the most suitable fit with parameters Burr(scale = 11.0, shape1 = 1.2, shape 2 = 0.9) (Fig. 5B). Based on the extrapolated distribution, we estimated the proportion of mortality cases below 6 months of age to be 0.77. This corresponds to a weight of 0.839 for cases before 6 months of age and a weight of 2.817 for 6 months and older.

To test the fit of the truncated distribution, we performed both a visual inspection (Fig. 5B) and a simulation test. We simulated data from the truncated distribution with the same sample size as the observed sample and calculated the median age. This was repeated a 1000 times. The median from the observed sample was compared with the distribution of medians from the simulated samples and a corresponding p-value was calculated. The permutation test did not yield a statistically significant result (p = 0.99), indicating that there is no compelling evidence that the fitted distribution is not representative of the true underlying age distribution.

### 3.4 Clinical characteristics

The metrics of the age profile (mean, median, and peak age) are presented in Table 1 for both the raw registry data and the raw prospective data (not adjusted for an overrepresentation of <6 months), and according to M1 and M2. Whereas the peak age of RSV-related mortality is consistent among the two models (driven by the prospective data, Table 1), M2 shows a younger age profile for the other metrics, with a median age difference of 2.2 months. Both models agree that the majority of RSV-related mortality cases from Gavi-eligible countries (59–77 %) occur before 6 months of life.

**Table 1:** Clinical characteristics of children under 5 years of age, who died with RSV in-hospital versus in the community in Gavi-eligible countries. Descriptive statistics (%, mean, medium, IQR) were calculated using survey weights, adjusting for the overrepresentation of studies with different age-related inclusion criteria. n is the reported frequency for a specific outcome in the dataset, N is the number of non-missing observations for the variable. n/N is the observed proportion in the database, not adjusted for survey weights.

Clinical characteristics	**Registry data** **N = 226**	Unadjusted prospective data* N = 197	**Complete data model (M1)** **N = 423**	**Prospective data model (M2)** **N = 197**
Sex, male, % (n)	52 (117/226)	48 (87/183)	51 (210/412)	47 (87/185)
Age at death, months, peak age (%)	2 (14)	1 (30)	1 (16)	1 (26)
Age at death, months, mean (SD)	6.0 (4.8)	2.9 (3.5)	5.9 (5.2)	4.4 (5.1)
Age at death, months, median (IQR)	4.9 (2.2–8.0)	1.7 (0.8–3.9)	4.3 (1.8–8.9)	2.1 (0.9–5.5)
Age <1 m at death, % (n)	7.5 (17)	30 (60)	14 (61)	26 (50)
Age <3 m at death, % (n)	30 (68)	66 (131)	37 (157)	56 (110)
Age <6 m at death, % (n)	60 (136)	92 (181)	59 (250)	77 (152)
Comorbidity, % (n)	37 (83)	18 (35)	31 (133)	30 (59)
Prematurity, % (n)	8.4 (19)	12 (23)	9.0 (38)	9.8 (19)
Hospitalized, % (n)	96 (217)	37 (73)	73 (311)	40 (79)
Gestational age (weeks) mean (SD)	37.1 (4.5)	35.9 (3.5)	36.4 (4.1)	37.0 (2.9)
n	13	16	32	25
Birth weight (kg) median (IQR)	2.6 (1.8–3.0)	2.8 (2.3–3.3)	2.8 (2.1–3.2)	2.9 (2.5–3.3)
n	22	31	59	53
Year of death, minimum–maximum	1995–2022	2017–2021	1995–2022	2017–2021
Not immunized, % (n/N)	12 (8/69)	53 (41/78)	26 (39/152)	39 (36/93)
Other children in household, % (n/N)	82 (28/34)	88 (29/33)	86 (64/75)	93 (44/47)
Mother uneducated, % (n/N)	21 (8/39)	11 (13/114)	15 (20/130)	11 (11/96)
Father uneducated, % (n/N)	0 (0/21)	4.0 (4/100)	3.2 (3/98)	4.0 (3/84)

* Not adjusted for the overrepresentation of cases <6 months of age.

Besides describing the age profile, our goal is to provide direct input for cost-effectiveness models. To accomplish this, we fitted a truncated Burr distribution to the M1 data for up to two years of age (Fig. 5A). Given that, for M2, the Burr distribution is fitted using information from below six months of age (Fig. 5B), we similarly utilized a truncated Burr distribution exclusively for the M1 data below six months. This approach ensures a fair comparison between the fitted distributions for both models and provides insight into the extent to which different age cut-offs impacted the fitted distribution. M2 yields a younger age-distribution compared to M1 (Fig. 5D). When only data below 6 months of age is used to fit M1, the age-distribution is still considerably younger compared to that of M2.

The q-q plots show a reasonably close alignment between the quantiles of the observed data and those predicted by the fitted distributions (SFigs. 3–6), except for the data fitted based on M2 up to two years of age. This is understandable, as the number of observations past 6 months are very limited in M2 and not evenly spread out (Fig. 5C). A visual comparison suggests a favorable fit of the distributions to the empirical data (Fig. 5A–C).

#### 3.4.1 Comorbidity and prematurity

Our estimations indicate that a minimum of 31 % and 30 % of mortality cases, for M1 and M2 respectively, had severe comorbidities (Table 1). Furthermore, based on M1 and M2, we calculated that a minimum of 9 % and 10 % of mortality cases, respectively, were born prematurely. Below 6 months of age, HIV/AIDS was the second most common reported comorbidity after congenital heart disease (STable 2). However, data on comorbidities and prematurity were often missing, especially for the Prospective Community Mortality Data cases (STable 3), limiting the power to analyze this characteristic. To address potential bias and test our assumption that children with missing data were born healthy term, we performed a sensitivity analysis excluding cases with missing data for prematurity or comorbidities. When excluding children with missing data for comorbidities and gestational age (STable 3), the percentage of children with comorbidities or prematurity more than doubled (to 59 % and 26 % for M1, and to 82 % and 27 % for M2, respectively).

#### 3.4.2 Community deaths vs in-hospital deaths

To address potential bias stemming from differences in data collection methodology (registry or prospective), a separate analysis was conducted for community and in-hospital deaths in both data collection approaches below 6 months of age (STable 4). Under 6 months of age, the observed age at time of death for in-hospital cases is older compared to community deaths. For both in-hospital cases and community deaths, the estimates for age at time of death are consistently older based on the Registry Mortality Data compared to the Prospective Community Mortality Data. We used a general linear model to compare the age at time of death (in months) for community deaths and in-hospital deaths below 6 months of age, while taking the differences in methodology into account. We found a significant effect of methodology on age at time of death, with the Prospective Community Mortality Studies resulting in lower estimates compared to the Registry Mortality Data (β = −0.7, p = 0.002), while the place of death (in community or in-hospital) showed no significant effect (β = 0.2, p = 0.35).

## 4 Discussion

To the best of our knowledge, this is the first global mortality study characterizing children dying with RSV specifically in Gavi-eligible countries. We found a peak age of RSV-related mortality at 1 month of age and the majority of RSV-related mortality cases from Gavi-eligible countries occur before 3–6 months of life. Thus, we expect that implementing infant RSV immunization strategies, such as maternal vaccination or infant immunoprophylaxis, will have high impact on RSV-related mortality in Gavi-eligible countries.

Impact and cost-effectiveness studies are required to further evaluate health and economic impact of RSV interventions for Gavi eligible countries. To inform these studies, we reported the fitted age at time of death distributions. We utilized a Burr distribution to model our data, as this continuous probability distribution is commonly employed to describe the duration of survival. Furthermore, the Burr-distribution is used as input for the UNIVAC model (step 2 of inputs page) in [13]); a decision-support model with a universal framework for evaluating the potential impact and cost-effectiveness of different vaccines. An adaptation of the UNIVAC model allows the evaluation of RSV maternal vaccines and infant mAbs [14], [15] and is therefore particularly useful in cost-effectiveness studies for RSV interventions.

We used two different models to estimate the age profile. The first model (M1) was based on all available data in the RSV GOLD database. The second model (M2) was based on only the data shared with the RSV GOLD project that were collected as part of the Prospective Community Mortality studies. Both M1 and M2 show a peak age of RSV-related mortality at 1 month of age. The majority of RSV-related mortality cases from Gavi-eligible countries occur before 6 months of life. According to M2, the majority of RSV-related mortality cases occur even before 3 months of life, suggesting a younger age-distribution compared to M1. The younger age profile of RSV mortality according to M2 is most in line with new available surveillance data. These contain thirty additional mortality cases from Gavi-eligible countries, gathered as part of the RSV GOLD ICU Network study, a recent active RSV surveillance study focusing on children under 2 years old, which have not been incorporated into the present analysis. The age profile of these cases is similar to that described in M2 [16].

On the other hand, the age distribution derived from M1 aligns more closely with previously described age distributions in the context of cost-effectiveness analyses in LMICs (SFig. 2). In a cost-effectiveness study conducted in Vietnam [15], a Burr-distribution was fitted to severe RSV-ALRI hospital admission data below 2 years of age. This distribution corresponds to a median age of 35 weeks. In another study by Mahmud et al. [14], data from RSV hospital admission in six LMICs were analyzed as part of their cost-effectiveness analysis. They identified the best fit for a Burr-distribution corresponding to a median age 23 weeks. Notably, their fitted age-distributions for severe RSV-ALRI exhibited significant variability across countries (see Fig. S1 in [14].). In both cost-effectiveness studies, the age-distribution for mortality cases was assumed to follow the age-distribution for hospital admission. However, this could result in an overestimation of the age-distribution, as mortality cases are generally younger [16]. This might explain why their estimates are notably older compared to our estimates.

For the first model (M1), it was assumed that using all available data except for the two community mortality studies, which had divergent age inclusion criteria, would provide us the most accurate representation of the true population. This assumption was based on two main factors: firstly, this data encompasses observations across the entire age spectrum up to 2 years, allowing for a comprehensive understanding of the age distribution below 2 years of age. Secondly, this is a relatively large and geographically diverse dataset. We acknowledge potential limitations in study methodology for the regular RSV GOLD registry cases; most of this data is gathered by our collaborators from hospital surveillance data or by systematically searching through hospital files. Heterogeneities in factors such as study setting, health-care access and seeking behaviour and eligibility for RSV testing could affect our estimates. For example, it has been speculated that younger children with RSV may present with nonspecific symptoms to the hospital and will therefore be overlooked [10]. Furthermore, children with reported severe comorbidities (who are generally older [9]) have better access to health care and health monitoring and therefore a higher probability of being tested and reported as RSV related death in the RSV GOLD database. Additionally, data pollution may arise due to the inclusion of children who died with severe comorbidities, where RSV was not a causal factor. These factors possibly explain the older age-distribution compared to M2.

For the second model, it was assumed that the subset of data obtained from the community mortality studies offers the most reliable depiction of the true population below 6 months of age. Unlike the regular GOLD registry cases, all these cases were collected through active surveillance. This active surveillance approach enhances the likelihood of capturing a comprehensive representation of the target population. An obvious limitation of this model is that the majority of the data originated from only one study site (Z-PRIME), and that it thereby makes the implicit assumption that the results of a single country are representative for all Gavi-eligible countries. Additionally, the reference data for this model only included children below 6 months of age. Therefore, M2 relies on the strong assumption that we can extrapolate the fitted distribution, obtained from data below 6 months, to up to 2 years of age.

Consistent with our previous analysis [10], we observed a lower median age at death in the community compared with in-hospital, although differences were not statistically significant when correcting for data collection methodology. Data collection methodology, on the other hand, was a significant predictor for age at time of death; with the Prospective Community Mortality Studies resulting in lower estimates compared to the Registry Mortality Data. This stresses the need for more high-quality prospectively collected mortality data from Gavi-eligible countries, to allow for more robust conclusions regarding the age profile of RSV mortality cases.

## 5 Conclusion

We expect that implementing infant RSV immunization strategies, such as maternal vaccination or infant immunoprophylaxis, will have high impact on RSV-related mortality in Gavi-eligible countries. We further conclude that the potential biases in retrospective and surveillance data influencing the age profile of RSV mortality cases should be considered when performing cost-effectiveness analyses and making policy decisions. We underscore the importance of collecting more high-quality prospectively collected mortality data from Gavi-eligible countries.

## Declaration of competing interest

The authors declare the following financial interests/personal relationships which may be considered as potential competing interests: LJB has regular interaction with pharmaceutical and other industrial partners. He has not received personal fees or other personal benefits. The University Medical Centre Utrecht (UMCU) has received major funding (>€100 000 per industrial partner) for investigator-initiated studies from AbbVie, MedImmune, Janssen, the Bill & Melinda Gates Foundation, Nutricia (Danone), and MeMed Diagnostics. UMCU has received major cash or in-kind funding as part of the public–private partnership IMI-funded RESCEU project from GSK, Novavax, Janssen, AstraZeneca, Pfizer, and Sanofi. UMCU has received major funding by GlaxoSmithKline and Julius Clinical for participating in the INFORM study sponsored by MedImmune. UMCU has received minor funding for participation in trials by Regeneron and Janssen from 2015 to 2017 (total annual estimate <€20 000). UMCU received minor funding for consultation and invited lectures by AbbVie, MedImmune, Ablynx, Bavaria Nordic, MabXience, Novavax, Pfizer, and Janssen (total annual estimate <€20 000). LJB. is the founding chairman of the ReSViNET Foundation. NIM has regular interaction with pharmaceutical and other industrial partners. She has not received personal fees or other personal benefits. Cheryl Cohen (C. C. has received minor funding from Sanofi and support to attend meetings from Parexel. Sandra Chaves (S. S. C.) has left the Centers for Disease Control and Prevention and is currently working for Sanofi Pasteur, France. All other authors report no potential conflicts. All authors have submitted the ICMJE Form for Disclosure of Potential Conflicts of Interest. Conflicts that the editors consider relevant to the content of the manuscript have been disclosed.

## Data Availability

Data will be made available on request.
