# Supplementary material for: Characteristics of inpatient and outpatient respiratory syncytial virus mortality in Gavi-eligible countries
Source: Vaccine X. 2024 Sep 13;20:100554. doi: 10.1016/j.jvacx.2024.100554 (PMC11417520; doi:10.1016/j.jvacx.2024.100554)
Supplement: Supplementary Data 1 [file mmc1.docx]

**Supplemental Materials**

**Supplemental methods**

In the Complete Data Model (M1), the population weights were determined using all available data except for the Z-PRIME community mortality study and the Pakistan data that had a different age of inclusion. We defined the proportion of cases that died before 6 months of age in the subset of data (i.e. all data excluding data from Z-PRIME and Pakistan) as P_<6*,_ and the proportion of cases that died before 6 months of age in the total dataset as P_<6_ (Step 1 and 2 in SFigure 1). The weights (W_<6_ and W_≥6_) were calculated based on the survey proportion (P_<6_) divided by the population proportion (P_<6*_), such that W_<6_ = P_<6_/ P_<6*_ and W_≥6_ = 1- W_<6_ (Step 3 in SFigure 1).

In the Prospective Data Model (M2), the population weights were estimated by using data from the Prospective Mortality Studies under 6 months of age only. We assumed that RSV-related childhood mortality follows a Burr distribution (type XII). This allowed us to fit a distribution to the truncated data and extrapolate the distribution. We fitted a truncated Burr distribution to the Prospective Mortality Studies Data below 6 months of age (Step 1, SFigure 1) using the “Burr” function from the “ExtDist” package[1] and the “optim” function from the “stats” package in R[2]. To extract the probability density before 6 months (N_<6_) and after 6 months (N_≥6_) from the distribution function up to two years of age, we again used the “Burr” function, taking into account the previously fitted parameters. The survey proportion was calculated based on the proportion of cases that died before 6 months of age in the Prospective Mortality Studies Data below 2 years (Step 2, SFigure 1). Again, the weights (W_<6_ and W_≥6_) were calculated based on the survey proportion (P_<6_) divided by the population proportion (P_<6*_), such that W_<6_ = P_<6_/ P_<6*_ and W_≥6_ = 1- W_<6_ (Step 3 in SFigure 1).

**References**

[1] Wu H, Godfrey AJR, Govindaraju K, Pirikahu S. Package ‘ExtDist’: Extending the Range of Functions for Probability Distributions. 2023; Available at: <https://cran.r-project.org/web/packages/ExtDist/ExtDist.pdf. Accessed 5 January 2024>.

[2] R Core Team (2022). R: A language and environment for statistical computing. R Foundation for Statistical Computing, Vienna, Austria. URL https://www.R-project.org/.


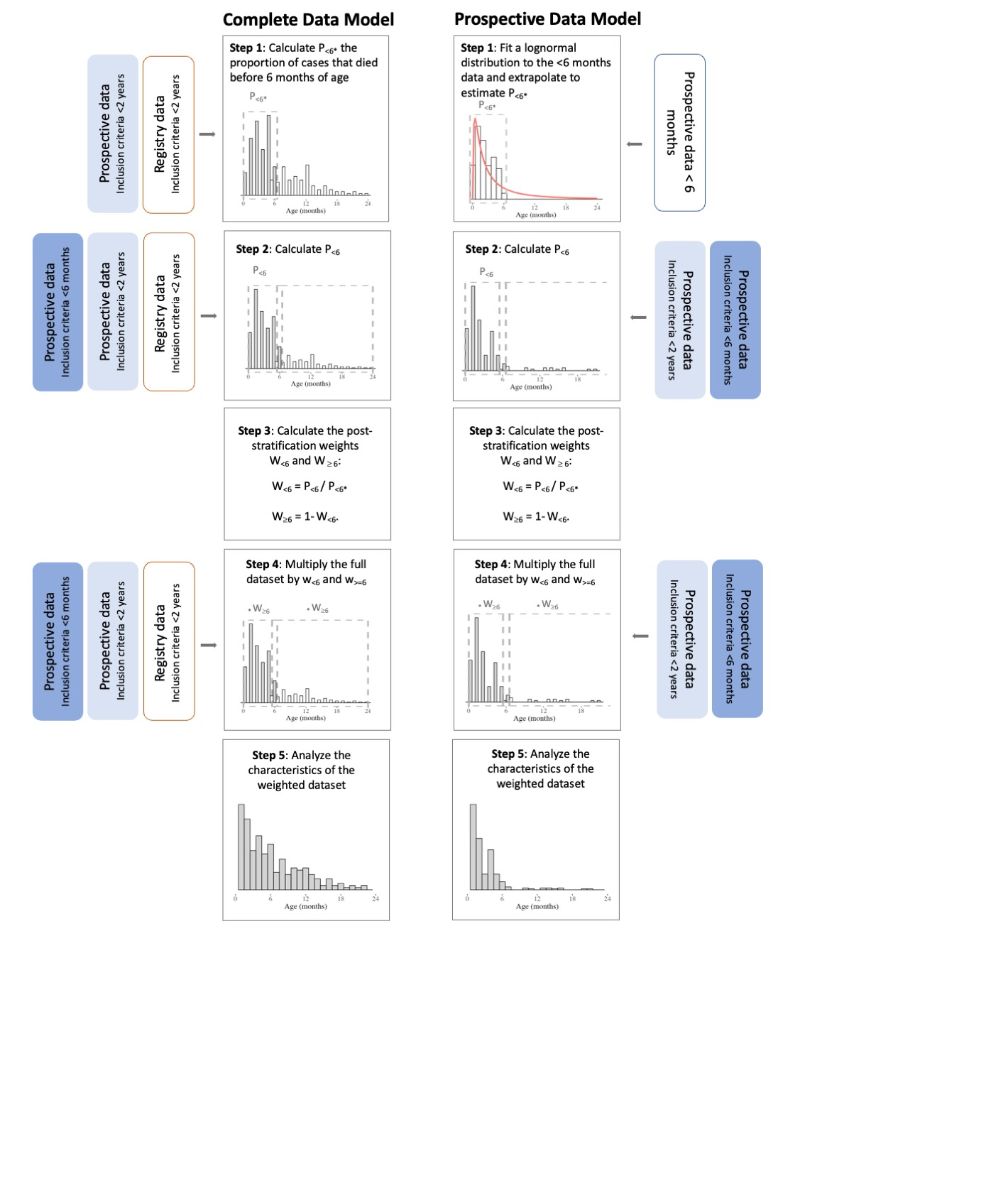


**Supplemental Figure 1:** Illustrative comparison depicting the disparities between the Prospective Data Model and the Complete Data Model.

**
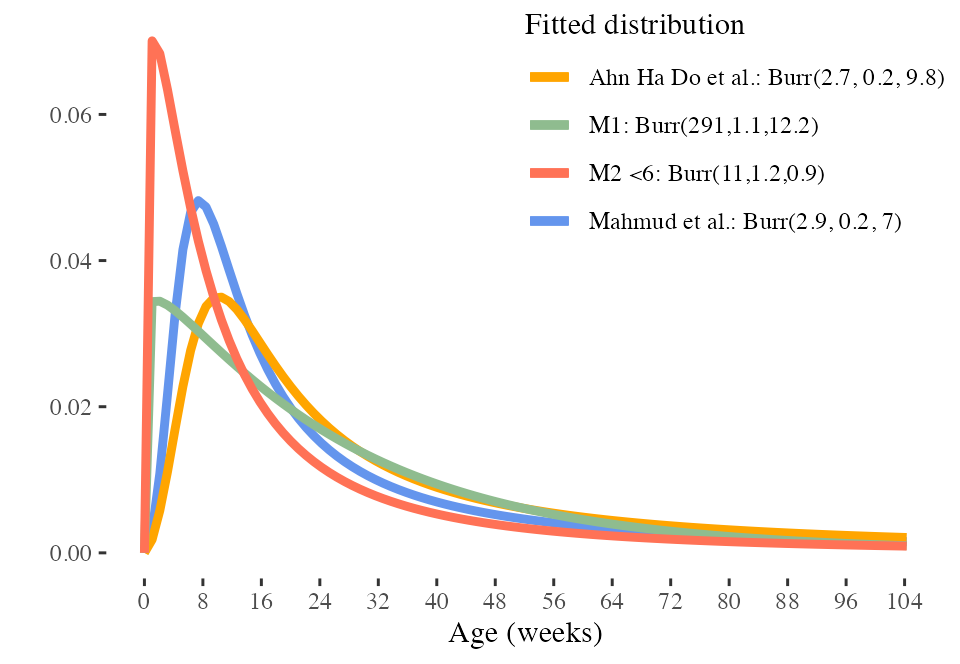
**

**Supplemental Figure 2:** Comparison of the fitted distributions for M1, M2, the distribution in Ahn Ha Do et al.[19] and Mahmud et al.[18]. Our fitted distributions (M1 and M2 <6) show a younger age at time of death distribution compared to the reported distributions in literature. The Burr distribution (Burr type XII) has three parameters: scale, shape 1 and shape 2. The fitted parameters are presented in the plot legend as Burr(scale, shape 1, shape 2). The cumulative distribution function of the Burr distribution (for *x* weeks of age) is $F\left( x \right)={1-[1+\left( \frac{x}{\mathrm{scale}} \right)^{shape 1}]}^{-shape 2}$.


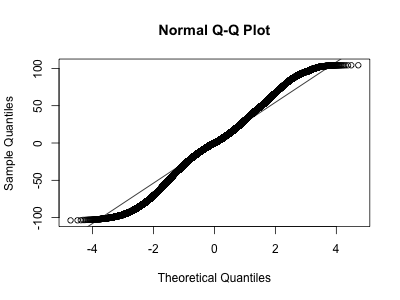


**Supplemental Figure 3**: **Q-Q plot M1**

Q-Q Plot of Fitted Burr-distribution on the observed weighted dataset according to M1.

***
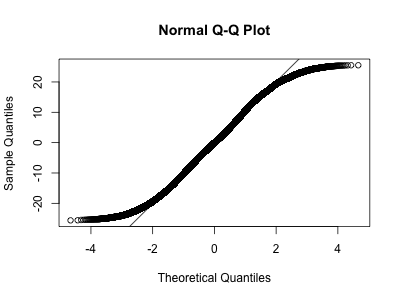
***

**Supplemental Figure 4**: **Q-Q plot M1 below 6 months**

Q-Q Plot of Fitted Burr-distribution on the prospective dataset below 6 months of age.

***
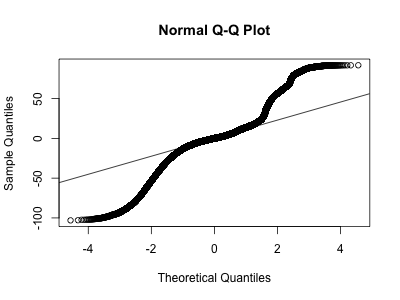
***

**Supplemental Figure 5**: **Q-Q plot M2**

Q-Q Plot of Fitted Burr-distribution on the observed weighted dataset according to M2.

***
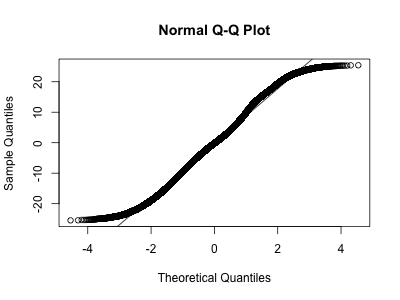
***

**Supplemental Figure 6**: **Q-Q plot M2 below 6 months**

Q-Q Plot of Fitted Burr-distribution on the complete dataset below 6 months of age

**STable 1. Origin of the data contributed to the RSV GOLD project and presented in this paper.**

N/A=Not applicable or not available.

| **Country** | **Number**  **of cases** | **Registry or prospective community study** | **PMID** | **Setting** | **Study period** | **Inclusion criteria** | **Exclusion criteria** | **Brief summary of methodology** |
| --- | --- | --- | --- | --- | --- | --- | --- | --- |
| Bangladesh | 9 | Registry | N/A | N/A | N/A | N/A | N/A | N/A |
| Bangladesh | 24 | Registry | 32017782 | Dhaka  hospital of International Centre for Diarrhoeal Disease Research | 2015-2017 | Severe acute malnourished (SAM) children <5 years admitted to the hospital | Children who might have a chance of migration within a one-month period from admission | Prospective case-control study with enrollment of SAM children admitted with clinical or radiological pneumonia (cases) and without any respiratory symptom in the 10 days prior to admission (controls). All nasopharyngeal wash material was tested for RSV. |
| Bangladesh | 20 | Registry | N/A | N/A | N/A | N/A | N/A | N/A |
| Burkina Faso | 2 | Registry | N/A | N/A | N/A | N/A | N/A | N/A |
| Burkina Faso | 1 | Registry | 32505586 | Saint Camille de Nanoro (rural area), hospitalized (Centre Medical avec Antenne chirurgicale (CMA)) and non-hospitalized children | 2016-2017 | Children with fever (temperature ≥38.0°C) or hypothermia (≤35.5°C), or a reported history of fever with signs of severe clinical illness or a suspicion of severe infection | Fever duration of >7 days | Prospective hospital-based diagnostic accuracy study. Enrolled children were tested for viral pathogens by nasal swabs or other diagnostic tests. From March until November 2016, only hospitalized children were enrolled. Thereafter, until the end of the study in June 2017, non-hospitalized children were also enrolled. |
| Gambia | 12 | Registry | 9535250 | 3 hospitals in the Western Region of Gambia. Hospitalized and non-hospitalized children | 1993-1996 | Children <2 years admitted to the hospitals, who presented with respirator infections | N/A | Data collection was partly prospective done for children enrolled in a vaccine trial. For the other children, data was collected retrospectively. Children were screened for RSV via nasopharyngeal aspirates. |
| Ghana | 1 | Registry | 30199549 | Korle Bu Teaching Hospital (tertiary hospital) and Princess Marie Louise Children’s Hospital (primary healthcare) | 2006, 2013-2014 | Children <5 years with acute lower respirator infection | Children with asthma and/or abnormal cardiovascular systems | Prospective enrollment of children and collection of nasopharyngeal aspirates or nasal swabs. Demographic and clinical information was collected on admission. |
| Kenya | 25 | Registry | 25712970 | Severe acute respirator illness (SARI) surveillance data from 8 countries | 2009-2012 | Adults and children >5 years, with SARI as defined by WHO recommendations | N/A | Information on all SARI cases and SARI-associated deaths detected by either systematic or non-systematic surveillance from 8 countries. Community deaths following discharge were not reported. |
| Kenya | 13 | Registry | N/A | N/A | N/A | N/A | N/A | N/A |
| Kenya | 59 | Registry | 20501927 | Hospital in a rural area: Kilifi District Hospital | 2007-2008 | Children aged 1 day to 12 years: i) admitted to the hospital meeting WHO clinical criteria for (very) severe pneumonia, ii) children with mild upper respiratory tract infection but not admitted to the hospital and iii) children attending for immunization | Children with severe respirator or cardiovascular disease | Prospective observational and case-control study during. After inclusion, a standardized set of investigations were performed, including nasal swabs. |
| Mali | 1 | Registry | 26696249 | Hospital in urban area, Gabriel Touré University Hospital | 2011-2012 | Children hospitalized with pneumonia | Children with presence of wheezing at auscultation | Prospective hospital-based case-control study. Cases were children with radiologically-confirmed pneumonia. Controls were patients hospitalized for surgery or in a routine outpatient practice environment, without any symptoms suggestive of respiratory illness. Cases and controls were matched for age and calendar date of hospital admission. |
| Mali | 1 | Registry | 32463443 | Home visits | 2011-2013 | Infants aged ≤6 months | N/A | Nested case-control study in a maternal influenza vaccine trial with 587 Malian mother-infant pairs, followed from birth to the age 6 months. RSV cases were infants who developed influenza-like illness (ILI) or pneumonia and were RSV-positive by PCR. Cases were matched to healthy controls and RSV-negative ILI controls. |
| Mozambique | 1 | Registry | N/A | N/A | N/A | N/A | N/A | N/A |
| Mozambique | 2 | Registry | 20805786 | Hospital in a rural area, Manhiça District Hospital | 2006-2007 | Children <5 years admitted to the hospital with signs or symptoms suggestive of clinical severe pneumonia | N/A | 1-year hospital-based surveillance with collection of a  nasopharyngeal aspirate |
| Mozambique | 3 | Registry | N/A | N/A | N/A | N/A | N/A | N/A |
| Multicountry: Ethiopia, Kenya, Mali, Mozambique, Sierra Leone | 33 | Prospective Community Study (CHAMPS) | 34472577 | 10 sites in 7 countries, both rural and urban | 2016-2019 | Children <5 years in the CHAMPS catchment area | Death reported >24 hours after death | Under-5 mortality surveillance in sub-Saharan Africa and South Asia, utilizing minimally invasive tissue sampling (MITS), postmortem laboratory and pathology testing, verbal autopsy and clinical and demographic data. |
| Multicountry: Kenya, Mali, Zambia | 13 | Registry | 31257127 | 9 sites in 7 countries | 2011-2014 | Children <5 years admitted to the hospital with severe pneumonia | Hospitalization within the preceding  14 days, having been discharged as a PERCH case within  the preceding 30 days, residence outside the study  catchment area and resolution of lower chest wall indrawing after  bronchodilator therapy for children with wheeze (for cases only) | Multisite, international case-control study. Controls were age-group-matched children randomly selected from communities surrounding study sites. |
| Nepal | 1 | Registry | 27241525 | Homebased surveillance for acute respirator illness (ARI) | 2011-2014 | Children aged <6 months | N/A | 2 consecutive, population-based, randomized, placebo-controlled trials were conducted in annual cohorts. Women (2nd or 3rd trimester) and their infants were followed with weekly home-based visits until 180 days after birth. Clinical and sociodemographic data were collected at each visit, and mid-nasal swabs were collected during ARI episodes. |
| Pakistan | 14 | Prospective Community Study (Pakistan) | 34472574 | Community-based mortality surveillance in 4 peri-urban settlements | 2018-2020 | Children ≤6 months and stillborns | N/A | Prospective community-based postmortem surveillance study. Nasopharyngeal swabs were collected within 24 hours after death. Home visits were conducted afterwards, and the WHO verbal and social autopsy tool was administered. |
| Pakistan | 3 | Registry | N/A | N/A | N/A | N/A | N/A | N/A |
| Pakistan | 1 | Registry | N/A | N/A | N/A | N/A | N/A | N/A |
| Pakistan | 3 | Registry | N/A | N/A | N/A | N/A | N/A | N/A |
| Togo | 1 | Registry | N/A | N/A | N/A | N/A | N/A | N/A |
| Uganda | 1 | Registry | 31306398 | 2 resource-limited hospitals: Jinja Regional Referral Hospital and Kambuga District Hospital | 2014-2015 | Children <13 years admitted to the hospital with signs of pneumonia | Children with clinically suspected tuberculosis | Prospective case-control study. Cases of RSV lower respiratory tract infection were defined as patients meeting the clinical inclusion criteria, plus detection of RSV from the nasopharynx by multiplex PCR. Controls were patients with rhinovirus respiratory tract infection and patients with suspected pneumococcal pneumonia. |
| Yemen | 9 | Registry | 28704440 | Sentinel SARI surveillance in 20 hospitals in 5 countries | 2007-2014 | Patients who met a syndromic case definition for SARI | N/A | Prospective sentinel SARI surveillance following a standardized methodology, using a standardized questionnaire and nasopharyngeal or oropharyngeal swabs |
| Yemen | 20 | Registry | 17073098 |  |  |  |  |  |
| Zambia | 150 | Prospective Community Study (ZPRIME) | 35063114 | Postmortem surveillance study in Lusaka, both in the community and hospitals | 2017-2020 | Infants 4 days – 6 months enrolled within 48 hours after death | N/A | Postmortem surveillance study. Demographic and clinical data were gathered from medical charts and death certificates and verbal autopsies were conducted. |

**STable 2. Frequency of reported comorbidities in Gavi-eligible countries of children who died with RSV before 6 months of age and after and including 6 months of age**.

|  | **In-hospital deaths** | | | **Community deaths** | | |
| --- | --- | --- | --- | --- | --- | --- |
|  | **<6 months** | **≥6 months** | **Total** | **<6 months** | **≥6 months** | **Total** |
|  | **n=182** | **n=96** | **n=278** | **n=135** | **n=10** | **n=145** |
| **Comorbidity,** % (n) | 57 (64/112) | 58 (42/73) | 57 (106/185) | 50 (7/14) | 100 (5/5) | 63 (12/19) |
| Congenital heart disease**,** % (n) | 24 (27/112) | 8.2 (6/73) | 18 (33/185) | 21 (3/14) | 0 (0/5) | 16 (3/19) |
| Chronic lung disease**,** % (n) | 0.9 (1/112) | 1.4 (1/73) | 1.1 (2/185) | 7.1 (1/14) | 0 (0/5) | 5.3 (1/19) |
| Immune disorder**,** % (n) | 2.7 (3/112) | 4.1 (3/73) | 3.2 (6/185) | 0 (0/14) | 0 (0/5) | 0 (0/19) |
| Genetic/chromosomal disease**,** % (n) | 8.9 (10/112) | 4.1 (3/73) | 7.0 (13/185) | 7.1 (1/14) | 0 (0/5) | 5.3 (1/19) |
| Down syndrome**,** % (n) | 4.5 (5/112) | 4.1 (3/73) | 4.3 (8/185) | 0 (0/14) | 0 (0/5) | 0 (0/19) |
| Neurological disease**,** % (n) | 2.7 (3/112) | 2.7 (2/73) | 2.7 (5/185) | 7.1 (1/14) | 20 (1/5) | 11 (2/19) |
| Airway abnormality**,** % (n) | 0.9 (1/112) | 1.4 (1/73) | 1.1 (2/185) | 0 (0/14) | 0 (0/5) | 0 (0/19) |
| Malaria**,** % (n) | 4.5 (5/112) | 11 (8/73) | 7.0 (13/185) | 7.1 (1/14) | 40 (2/5) | 16 (3/19) |
| HIV/AIDS**,** % (n) | 7.1 (8/112) | 14 (10/73) | 9.7 (18/185) | 14 (2/14) | 40 (2/5) | 21 (4/19) |
| Tuberculosis**,** % (n) | 0.9 (1/112) | 6.8 (5/73) | 3.2 (6/185) | 0 (0/14) | 0 (0/5) | 0 (0/19) |
| Malignancy**,** % (n) | 0 (0/112) | 1.4 (1/73) | 0.5 (1/185) | 0 (0/14) | 0 (0/5) | 0 (0/19) |
| Other comorbidity**,** % (n) | 9.8 (11/112) | 11 (8/73) | 10 (19/185) | 7.1 (1/14) | 100 (5/5) | 32 (6/19) |
| **Prematurity,** % (n) | 36 (28/78) | 9.4 (3/32) | 28 (31/110) | 31 (8/26) | 38 (3/8) | 32 (11/34) |

**STable 3. Clinical characteristics of children under 6 months who died with RSV in-hospital versus in the community in Gavi-eligible countries, without making assumptions about missing data**. n is the reported frequency for a specific outcome in the dataset, N is the number of non-missing observations for the variable. n/N is the observed proportion in the database, not adjusted for survey weights.

| Clinical Characteristics | **Registry data**  **N = 226** | **Unadjusted Prospective Data***  **N = 197** | **Complete Data Model (M1)**  **N = 423** | **Prospective Data Model (M2)**  **N = 197** |
| --- | --- | --- | --- | --- |
| Comorbidity, % (n/N) | 53 (83/156) | 73 (35/48) | 59 (133/227) | 82 (59/72) |
| Prematurity, % (n/N) | 24 (19/80) | 36 (23/64) | 26 (38/147) | 27 (19/72) |
| Hospitalized, % (n/N) | 96 (217/226) | 37 (73/195) | 73 (308/421) | 39 (75/193) |

**STable 4. Clinical characteristics of children under 6 months who died with RSV in-hospital versus in the community in Gavi-eligible countries, for the Prospective Mortality Data and Registry Mortality Data seperately.** Descriptive statistics (%, mean, medium, IQR) were calculated using survey weights, adjusting for the overrepresentation of studies with different age-related inclusion criteria. n is the reported frequency for a specific outcome in the dataset, N is the number of non-missing observations for the variable. n/N is the observed proportion in the database, not adjusted for survey weights.

|  | <6 months, prospective mortality studies | | <6 months, registry data | | |
| --- | --- | --- | --- | --- | --- |
|  | **Community deaths** | **In-hospital deaths** | | **Community deaths** | **In-hospital deaths** |
|  | **n=124** | **n=57** | | **n=11** | **n=125** |
| Sex, male, % (n) | 54 (59/110) | 37 (21/57) | | 55 (6/11) | 48 (60/125) |
| Age at death, months, peak age (%) | 1 (35) | 1 (28) | | 2 (45) | 2 (21) |
| Age at death, months, mean (SD) | 2.0 (1.6) | 2.2 (1.6) | | 2.7 (1.4) | 2.9 (1.6) |
| Age at death, months, median (IQR, n) | 1.4 (0.7-3.3) | 1.9 (0.8-3.5) | | 2.0 (1.9-3.5) | 3.0 (1.8-4.0) |
| Age <1 m at death, % (n) | 34 (42) | 32 (18) | | 9.1 (1) | 13 (16) |
| Age <3 m at death, % (n) | 74 (92) | 68 (39) | | 64 (7) | 49 (61) |
| Comorbidity, % (n) | 4.8 (6) | 25 (14) | | 9.1 (1) | 40 (50) |
| Prematurity, % (n) | 4.8 (6) | 30 (17) | | 18 (2) | 8.8 (11) |
| Hospitalized, % (n) | 8.9 (11) | 93 (53) | | 36 (4) | 100 (125) |
| Year of death, minimum - maximum | 2018-2020 | 2018-2020 | | 2009-2022 | 1995-2022 |
